# *Bordetella pertussis* Infection or Vaccination Substantially Protects Mice against *B. bronchiseptica* Infection

**DOI:** 10.1371/journal.pone.0006778

**Published:** 2009-08-26

**Authors:** Elizabeth M. Goebel, Xuqing Zhang, Eric T. Harvill

**Affiliations:** 1 Department of Veterinary and Biomedical Sciences, the Pennsylvania State University, University Park, Pennsylvania, United States of America; 2 Graduate Program in Immunology and Infectious Diseases, the Pennsylvania State University, University Park, Pennsylvania, United States of America; 3 Graduate Program in Genetics, the Pennsylvania State University, University Park, Pennsylvania, United States of America; Charité-Universitätsmedizin Berlin, Germany

## Abstract

Although *B. bronchiseptica* efficiently infects a wide range of mammalian hosts and efficiently spreads among them, it is rarely observed in humans. In contrast to the many other hosts of *B. bronchiseptica*, humans are host to the apparently specialized pathogen *B. pertussis*, the great majority having immunity due to vaccination, infection or both. Here we explore whether immunity to *B. pertussis* protects against *B. bronchiseptica* infection. In a murine model, either infection or vaccination with *B. pertussis* induced antibodies that recognized antigens of *B. bronchiseptica* and protected the lower respiratory tract of mice against three phylogenetically disparate strains of *B. bronchiseptica* that efficiently infect naïve animals. Furthermore, vaccination with purified *B. pertussis*-derived pertactin, filamentous hemagglutinin or the human acellular vaccine, Adacel, conferred similar protection against *B. bronchiseptica* challenge. These data indicate that individual immunity to *B. pertussis* affects *B. bronchiseptica* infection, and suggest that the high levels of herd immunity against *B. pertussis* in humans could explain the lack of observed *B. bronchiseptica* transmission. This could also explain the apparent association of *B. bronchiseptica* infections with an immunocompromised state.

## Introduction

The emergence of new infectious diseases from zoonotic sources may be constrained by at least three limiting steps. First, a pathogen must have the opportunity, observed when organisms “spillover” from their natural host to humans. Second, it must have the ability to colonize/infect an individual and to expand in numbers there. Third, it must successfully spread to new human hosts and establish a successful chain of transmission [1], [2]. It is important to consider the factors that can affect each of these steps in order to understand the risk of newly emerging diseases. Successful transmission requires shedding of the pathogen from an infected individual and contact with susceptible individuals. In the simplest case of a novel infectious agent, all of a new host population are often considered to be susceptible until they are infected, recover and may then be immune, at least for a time. However, the susceptibility of individual hosts to a novel agent may be affected by various factors, including the resident flora and immunity to it. Since pathogen control strategies such as vaccination can change the immune status of individuals, and the human population, it is important to understand how that might affect their susceptibility to not just that pathogen, but to other closely related pathogens that are likely to spillover from animal sources.

The classical bordetellae are closely related gram-negative bacterial species which colonize the respiratory tracts of a variety of mammalian hosts [3]. *Bordetella bronchiseptica* causes a chronic respiratory infection that can persist for the life of the animal [3], [4]. This pathogen has been isolated from a diverse range of mammalian hosts and is associated with kennel cough in dogs, snuffles in rabbits and atrophic rhinitis in pigs but may be present without symptoms in a majority of these and other animals [3]–[5]. The individual lineages of *B. bronchiseptica* do not appear to be host-specific, since very closely related strains have been shown to efficiently infect a wide range of mammals, including humans [5]–[16]. Although dogs and other animals are common hosts for a wide variety of lineages of *B. bronchiseptica*, one specialized lineage of *Bordetella* has adapted to occupy humans as its only apparent ecological niche; *B. pertussis* is prevalent worldwide as a highly infectious pathogen that causes the acute disease whooping cough [3]. Although infections are cleared by host immunity, *B. pertussis* persists within human populations by reinfecting previously infected or vaccinated individuals [3], [17]. We have suggested that the very high prevalence of antibodies to *B. pertussis*, detectable in greater than 90% of individuals, may reflect immunity that affects the emergence of closely related pathogens [17].

Other classical bordetellae retain the ability to infect humans, but only *B. parapertussis* appears to successfully occupy humans as a primary ecological niche [3], [18]. Since immune-mediated competition would be expected to exclude one or the other from a shared host population, we hypothesized, and recently showed, that *B. parapertussis* avoids cross-immunity via the expression of an O-antigen not shared by *B. pertussis* which prevents the binding of antibodies induced by the latter to shared antigens on the bacterial surface [13], [15], [19]–[21]. This appears to have allowed *B. parapertussis* to invade a human population in which *B. pertussis* was already endemic.

Animals in very close contact with humans, such as dogs and cats, are known to be infected with a very wide range of different *B. bronchiseptica* strains, which are highly infectious and spread amongst them rapidly. Interestingly, a similarly broad set of *B. bronchiseptica* strains have been occasionally isolated from humans, indicating they retain the ability to infect humans, but they generally are not observed to spread between humans [5], [12]. The observations that *B. bronchiseptica* can spillover to humans from animals, and can infect and cause disease, particularly in immunocompromised individuals, led us to explore how the infectious process may be affected by immunity to the resident pathogen, *B. pertussis*.

Here we quantified the effects of *B. pertussis*-induced immunity on the ability of *B. bronchiseptica* to successfully infect mice [22]–[24]. We show that *B. pertussis* infection- and vaccine-induced immunity protects against *B. bronchiseptica* colonization in the lower respiratory tract (LRT). This protection, defined as a significant decrease in bacterial load in the LRT of an immunized host, appears to be mediated by cross reacting antibodies which recognize shared antigens. Immunization with the *B. pertussis*-derived antigens in the acellular vaccine, Adacel, or the individual proteins pertactin or filamentous hemagglutinin, was sufficient to induce protective immunity to *B. bronchiseptica*. Thus *B. pertussis* vaccination- or infection-induced immunity can protect the LRT of mice against *B. bronchiseptica*. Together with our previous demonstration that *B. parapertussis* avoids *B. pertussis*-induced immunity, these data may explain, in whole or in part, why *B. bronchiseptica* is observed to spillover to humans from other animals in which it causes epidemics, but is generally associated with disease only in immunocompromised humans.

## Materials and Methods

### Bacterial Growth

*Bordetella pertussis* strain 536, a streptomycin resistant derivative of Tohama I [25], *B. bronchiseptica* strain RB50 [26], and *B. bronchiseptica* strain RB50G, a gentamicin-resistant derivative of RB50 [27], have been previously described. Human isolates of *B. bronchiseptica*, strains A345 (a.k.a. B2493 and GA96-01) and M0149 (a.k.a. D444 and B2494), were received from the CDC and multi-locus sequence typed as previously described [5]. All strains were maintained on Bordet-Gengou (BG) Agar (Difco, Sparks, MD) with 10% sheep's blood (Hema Resources, Aurora, OR) with 20 µg/ml of streptomycin or gentamicin. Bacteria were grown overnight at 37°C in Stainer-Scholte broth [28]–[30] to mid-exponential phase and diluted in phosphate buffered saline (PBS, Omnipur, Gibbstown, NJ) to the indicated concentration.

### Animal Care and Housing

4 to 6 week old C57BL/6, µMT and RAG2 ^−/−^ mice were obtained from Jackson Laboratories (Bar Harbor, ME). IgA ^−/−^ mice were obtained from Dr. Innocent Mbawuike [31]. C3 ^−/−^ mice were obtained from Dr. Rick Wetsel [32]. Mice lacking Fcγ Receptor I, II, and III (FcγR^−/−^) were obtained from Taconic (Hudson, NY). All mice were maintained and bred in Pennsylvania State University approved housing facilities and were closely monitored in accordance with institutional policies and the Institutional Animal Care and Use Committee (IACUC) regulations (IACUC approval number for breeding: 31180 and for experiments: 31297).

### Inoculation and Vaccination of Mice

For inoculation, mice were lightly sedated by a flow of 5% isoflurane in oxygen and a 50 µl inoculum containing 5×10^5^ CFU was pipetted gently onto the external nares. This method of inhalation inoculation reliably distributes the bacteria throughout the respiratory tract [22]. For vaccination, the mice were intraperitoneally (i.p.) injected with 1×10^8^ heat killed *B. pertussis* in 1 ml of PBS [33], 40 µg of purified pertactin 1 [34] or 5 µg of filamentous hemagglutinin (Sigma, St. Louis, MO) in 200 µL of PBS with Imject Alum (Pierce, Rockford, IL), or subcutaneously injected with 0.5 ml of 1∶5 dilution of the 5-component human vaccine, Adacel (aP) (Sanofi-Pasteur, Swiftwater, PA) in PBS with Imject Alum on Day 0 and Day 14. Mice were euthanized by CO_2_ inhalation and nasal cavities, trachea and lungs were excised, homogenized and serially diluted in PBS. Aliquots were then plated on BG Agar with appropriate antibiotics and the resultant colonies were counted two days later.

### Antibiotic Treatment and Depletion of Immune Factors

For all reinfection experiments, 1% gentamicin (Sigma, St. Louis, MO) was administered in the drinking water for 3 days beginning on day 23 post-*B. pertussis* inoculation [35]. Mice were then given untreated water for 2 days prior to challenge with RB50G. Previous studies have shown that gentimicin treatment does not hinder *B. bronchiseptica* strain RB50G colonization of the murine respiratory tract [35]. Neutrophils were depleted by i.p. injection of 1 mg of mAb from the hybridoma RB6-8C5 (αLy-6G) 48 hours prior to bacterial inoculation [33]. Depletion of 95% of blood neutrophils was confirmed via CBC differential count. Complement was depleted by two i.p. injections of 5 units of Cobra Venom Factor (CVF, Sigma, St. Louis, MO) 24 and 22 hours prior to inoculation [36].

### Generation and Passive Transfer of Immune Serum

Convalescent mice were generated by inoculating mice with the indicated bacteria and allowing the mice to recover for 28 days [33]. Pooled serum was collected via post-mortem cardiac puncture from wild type convalescent or naive mice. To induce a higher titer of *B. pertussis*-specific antibodies, *B. pertussis*-inoculated mice were allowed to convalesce for 28 days followed by a second challenge with *B. pertussis* and subsequent cardiac puncture 3 days post secondary inoculation. To generate complement deficient serum, sera were heat-inactivated at 65°C for 30 minutes prior to passive transfer. Passive transfer experiments used 200 µL of serum i.p. injected at the time of inoculation [23], [33].

### Western Blots Analysis

Westerns Blots were performed on lysates of 1×10^6^ CFU of *B. pertussis,* or *B. bronchiseptica* in 10 µL Laemmli sample buffer (Bio-Rad, Hercules, CA). Lysates were run on 7% SDS-PAGE gels under denaturing conditions and then transferred to PVDF membranes. The membranes were then probed overnight with a 1∶100 dilution of serum collected from a naïve mouse or a mouse inoculated with a single dose of *B. pertussis* for 28 days (5×10^5^ CFU in 50 ul) and a 1∶10,000 dilution of goat anti-mouse Ig (H+L) HRP-conjugated (Southern Biotech, Birmingham, AL) was used as the detector antibody. The membrane was visualized with ECL Western Blotting detection reagents (Amersham Biosciences, Piscataway, NJ).

### Statistical Analysis

Student's two-tailed t-test was used to determine statistical significance between experimental groups. *P*-values of≤0.05 were considered significant. Error bars represent SEM.

## Results

### Immunity to *B. pertussis* protects against *B. bronchiseptica* challenge in the LRT

**Figure 1:**
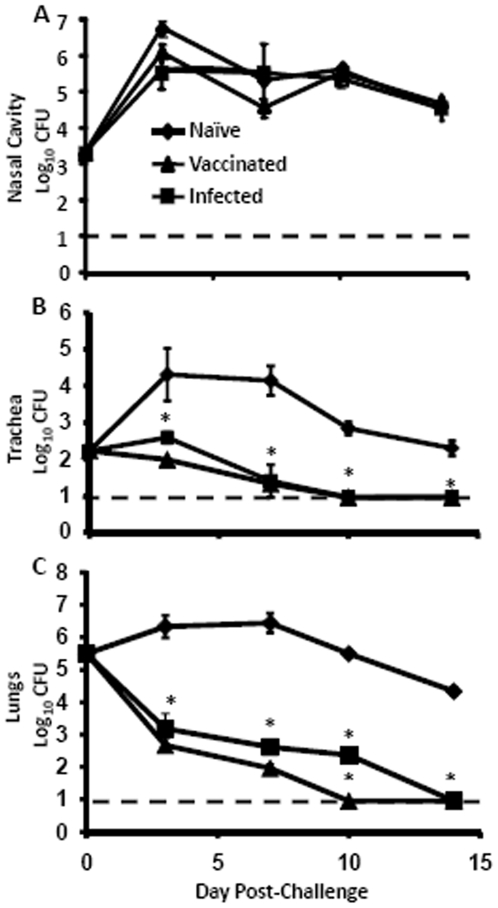
*B. bronchiseptica* numbers in the respiratory tract of naïve and *B. pertussis* immune mice over time. Groups of 3 to 4 C57BL/6 mice were left uninoculated (♦), inoculated with 5×10^5^ CFU of *B. pertussis* (▪), or vaccinated with 2 i.p. injections of 10^8^ heat killed *B. pertussis* on Days 0 and 14 (▴). On Day 21, mice were gentimicin-treated for 3 days. Mice were then challenged with a gentimicin^R^ strain of *B. bronchiseptica* on Day 28 and sacrificed at the indicated day post-challenge for quantification of bacterial load in nasal cavity (A), trachea (B) and the lungs (C). Bacterial numbers are represented as the mean Log_10_ CFU+/−SEM. Dashed line represents the lower limit of detection. * indicates statistical difference (*P* value<0.05) between naïve and treated groups.

To test the hypothesis that *B. pertussis*-induced immunity protects against *B. bronchiseptica* colonization of the LRT, wild type mice were inoculated with live *B. pertussis* or vaccinated with heat-killed *B. pertussis* and allowed to recover for at least 28 days. By this time point, bacterial numbers in the respiratory tract were reduced to approximately 10^2^ or fewer CFU and a strong immune response had been induced [23]. *B. pertussis*-vaccinated, -previously infected, or naïve mice were gentamicin treated to clear any remnant bacteria and then challenged with a gentamicin resistant strain of *B. bronchiseptica* and the bacterial load was enumerated on Days 3, 7, 10 and 14 post-challenge. Approximately 10^4^ CFU in the trachea and 10^6^ CFU in the lungs were recovered on both Days 3 and 7 post-challenge in naïve mice (Figure 1). However, only approximately 1% (10^2^ CFU) and 0.05% (10^3^ CFU) *B. bronchiseptica* were recovered from the trachea and lungs by Day 3 post-challenge in immunized mice as compared to naïve animals (Figure 1). By Day 7 post-challenge, previously infected mice retained approximately 10^2^ CFU of *B. bronchiseptica* in the trachea, and 10^3^ CFU of *B. bronchiseptica* in the lungs, and carried these low loads of *B. bronchiseptica* for approximately 2 weeks, until bacteria were undetectable on Day 14 post-challenge. Vaccinated mice cleared *B. bronchiseptica* from the trachea and lungs by Day 7 post-inoculation (Figure 1). These data support the hypothesis that immunity to *B. pertussis* protects against a *B. bronchiseptica* challenge in the LRT. In the nasal cavities, *B. pertussis* immunized mice have similar colonization kinetics to naïve mice (Figure 1). Although immunity to *B. pertussis* has limited effects on *B. bronchiseptica* colonization in the nasal cavity, the rapid and significant reduction of *B. bronchiseptica* numbers in the LRT of *B. pertussis* immunized hosts minimized the inflammation and pathology which would have further led to disease symptoms and transmission. Because a very large and significant effect of prior exposure to *B. pertussis* on the colonization of *B. bronchiseptica* was observed on Day 3 post-challenge, subsequent experiments were carried out at this timepoint.

### *B. pertussis*-induced immunity requires B cells, but not IgA, for protection against *B. bronchiseptica* challenge in the lungs

**Figure 2:**
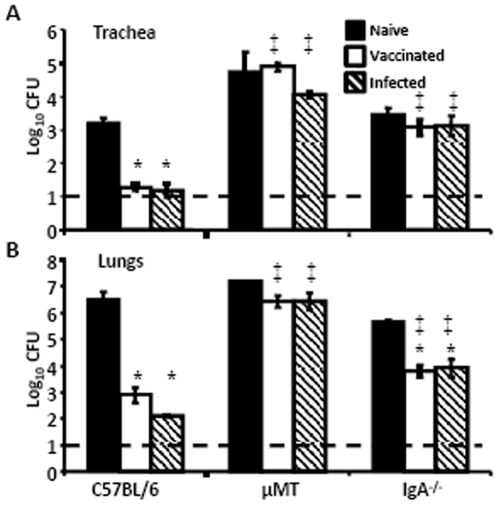
*B. bronchiseptica* numbers in the LRT of immunized B-cell and IgA deficient mice. Naïve (black bars), *B. pertussis*-vaccinated (white bars), or *B. pertussis*-convalescent (hatched bars) C57BL/6, µMT and IgA^−/−^ mice were dissected Day 3 post gentimicin^R^ *B. bronchiseptica* challenge. Bacterial numbers in the trachea (A) and lungs (B) are represented as the mean Log_10_ CFU+/−SEM. Dashed line indicates the lower limit of detection. * indicates statistical difference (*P* value<0.05) between naïve and treated groups. ‡ indicates statistical difference (*P* value<0.05) between mutant mouse and similarly treated wild type mouse groups.

Since immunization with *B. pertussis* led to protection against *B. bronchiseptica*, we sought to determine if this was mediated by the adaptive immune response. *B. pertussis*-immunized RAG^−/−^ mice were unable to reduce *B. bronchiseptica* numbers, indicating a role for adaptive immunity in protection (data not shown). We hypothesized that *B. pertussis*-induced antibodies could recognize *B. bronchiseptica* antigens and mediate the clearance of this pathogen. Supporting this hypothesis, there was no difference in bacterial load in the trachea or the lungs between immunized or naïve µMT mice on Day 3 post *B. bronchiseptica* challenge (Figure 2). These data indicate that B cells, and the antibodies they produce [23], are required for cross protection.

We further hypothesized that the major mucosal antibody, IgA, would be the primary protective antibody [35]. IgA^−/−^ mice were vaccinated or infected with *B. pertussis* and allowed to convalesce for 28 days. The treated or naïve mice were then challenged with *B. bronchiseptica* and dissected Day 3 post-inoculation. The bacterial load in the lungs of naïve IgA^−/−^ mice was not significantly different from naive wild type mice (Figure 2). Consistent with previous findings, immunized IgA^−/−^ mice were not protected against *B. bronchiseptica* challenge in the trachea [35]. Immunization reduced *B. bronchiseptica* numbers in the lungs of IgA−/− mice by greater than 95%, although immunization reduced bacterial numbers by >99.9% in wild type mice, relative to naïve mice. Thus, IgA is required for *B. pertussis*-immune-mediated protection against *B. bronchiseptica* in the trachea, but is not as important in the lungs.

### *B. pertussis*-induced sera require complement and neutrophils to reduce *B. bronchiseptica* colonization

**Figure 3:**
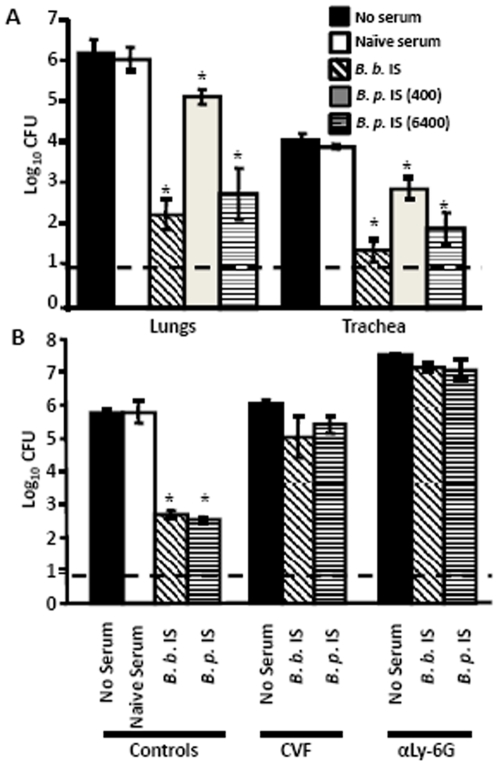
Effect of Complement and Neutrophils on *B. pertussis* serum antibody-mediated clearance of *B. bronchiseptica*. (A) Wild type mice infected with *B. bronchiseptica* were untreated (black bars) or i.p. injected with naïve serum (white bars), *B. bronchiseptica*-immune serum (hatched bars), *B. pertussis*-immune serum titer 400 (gray bars), or *B. pertussis*-immune serum titer 6400 (horizontal lined bars) and then dissected Day 3 post-inoculation. (B) Wild type mice were complement (CVF) or neutrophil (αLy-6G) depleted, then inoculated with *B. bronchiseptica*, i.p. injected with the indicated serum and then dissected on Day 2 post-inoculation. *B.b*. indicates *B. bronchiseptica*-immune serum; *B.p*. indicates *B. pertussis*-immune serum titer 6400. Bacterial numbers are represented as the mean Log_10_ CFU+/−SEM. Dashed line indicates the lower limit of detection. * indicates *P* value of <0.05.

To determine if *B. pertussis*-induced sera can clear *B. bronchiseptica* upon passive transfer into naïve mice, wild type mice were inoculated with *B. bronchiseptica* and immediately i.p. injected with 200 µL of naïve, *B. bronchiseptica-* or *B. pertussis*-induced immune serum. The mice were then dissected on Day 3 post-inoculation for bacterial enumeration. Compared to untreated mice, naïve serum had no effect on bacterial numbers (Figure 3A). In contrast, *B. pertussis*-immune serum (titer ∼400) reduced bacterial numbers in the lungs and trachea by greater than 85% (Figure 3A). *B. pertussis*-immune serum with a titer of 6400, a titer similar to that of *B. bronchiseptica*-induced immune serum, reduced *B. bronchiseptica* numbers in both organs by>99.9% relative to that of naïve serum treated mice, an effect similar to that of *B. bronchiseptica*-immune serum (Figure 3A). Together, these data suggest that *B. pertussis*-induced sera are able to efficiently reduce numbers of *B. bronchiseptica* in the LRT in a dose-dependent manner. Naïve serum, while containing other components such as albumin and complement, had no effect on bacterial numbers, suggesting *B. pertusis*-specific antibodies mediate the reduction of *B. bronchiseptica* colonization.

Our previous work showed that antibodies induced by *B. bronchiseptica* infection clear this bacterium from the lungs of mice via a complement and neutrophil-dependent mechanism [37], [38]. Thus, we hypothesized that *B. pertussis*-induced antibodies would also required the complement cascade and/or neutrophils in order to clear *B. bronchiseptica*. To test this hypothesis, mice were CVF treated to deplete complement or were treated with αLy-6G monoclonal antibody to deplete neutrophils prior to inoculation with *B. bronchiseptica*. While *B. pertussis*-immune serum reduced *B. bronchiseptica* numbers in untreated mice, it did not significantly reduce bacterial numbers in the lungs of CVF or αLy-6G treated mice, indicating that both complement and neutrophils are required (Figure 3B). In addition, all αLy-6G treated mice were moribund by Day 2 post-inoculation, indicating that *B. pertussis*-specific sera did not protect against the rapid virulence of *B. bronchiseptica* in animals lacking neutrophils.

**Figure 4:**
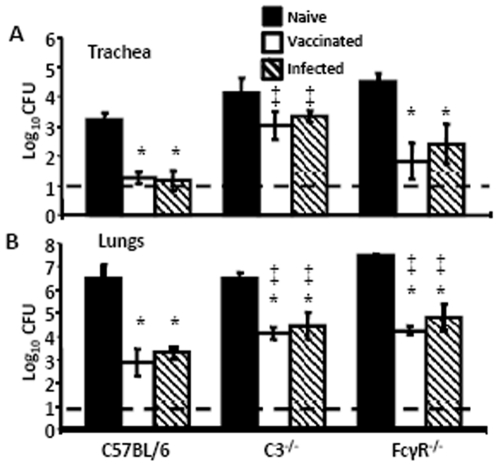
Clearance of *B. bronchiseptica* from the LRT in *B. pertussis*-immunized C3^−/−^ and FcγR^−/−^ mice. Naïve (black bars), *B. pertussis*-vaccinated (white bars), or *B. pertussis*-convalescent (hatched bars) mice were dissected Day 3 post gentimicin^R^ *B. bronchiseptica* inoculation. Bacterial numbers in the (A) trachea and (B) lungs are represented as the mean Log_10_ CFU+/−SEM. Dashed line indicates the lower limit of detection. * indicates statistical difference (*P* value<0.05) between naïve and treated mouse. ‡ indicates statistical difference (*P* value<0.05) between mutant and similarly treated wild type mice.

To determine if complement and Fcγ Receptors are required for *B. pertussis*-immunization induced protection of the LRT against *B. bronchiseptica*, C3^−/−^ mice, which lack the enzyme required for both the classical and the alternative complement cascades, and FcγR^−/−^ mice, which lack Fcγ Receptors (I, II, and III) specific for the Fc region of IgG antibodies, were immunized with *B. pertussis* and then challenged 28 days later with *B. bronchiseptica*. In both C3^−/−^ and FcγR^−/−^ mice that were previously vaccinated or infected with *B. pertussis*, the *B. bronchiseptica* bacterial load in the lungs was significantly decreased by Day 3 post-inoculation when compared to naïve mice (Figure 4), indicating that either vaccination or infection can confer protection in the absence of either C3 or FcγRs. In the trachea, *B. pertussis* immunization reduced bacterial numbers significantly in FcγR^−/−^ mice, but not significantly in C3^−/−^ mice, relative to naïve mice (Figure 4). Together these results are consistent with our previous findings that adoptively transferred immune serum conferred protection against *B. bronchiseptica* that was dependent on both complement and FcγRs (Figure 3)[33], but infection-induced protection was much less dependent on either (Figure 4)[39].

### *B. pertussis*-induced sera recognize antigens on *B. bronchiseptica*

**Figure 5:**
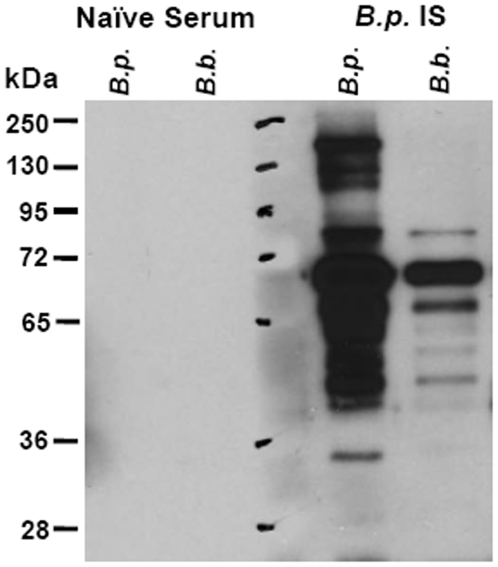
Analysis of cross-reacting antigens between *B. pertussis* and *B. bronchiseptica*. Lysates of *B. pertussis* (*B.p.*) or *B. bronchiseptica* (*B.b.*) were loaded in the indicated wells and separated by SDS-PAGE gel. Proteins were transferred to PVDF membrane, and then probed with naïve serum or *B. pertussis*-infection induced immune serum (*B.p.* IS).

Since passive immunization with *B. pertussis*-induced sera can control *B. bronchiseptica* infection in the LRT, we examined recognition of *B. bronchiseptica* antigens by *B. pertussis*-infection-induced serum. Western Blot analysis of *B. pertussis* and *B. bronchiseptica* lysates probed with *B. pertussis*-infection-induced serum showed that *B. pertussis*-infection-induced serum antibodies were cross reactive with *B. bronchiseptica* antigens (Figure 5). The size of one of the cross-reactive bands at approximately 65-72 KDa suggested that the band was pertactin, an antigenic protein known to induce protective immunity [24], [34], [40]. Several other *B. bronchiseptica* antigens appeared to be cross-reactive to *B. pertussis*-infection-induced serum. There was little to no recognition of these lysates when probed with naïve serum at the same dilution.

### Immunization with *B. pertussis*-derived antigens is protective against *B. bronchiseptica* challenge

**Figure 6:**
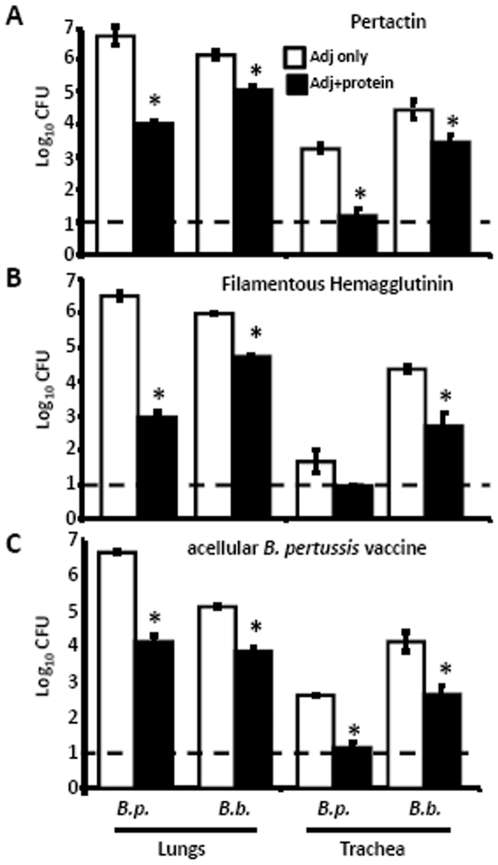
Effect of vaccination with *B. pertussis*-derived antigens on *B. bronchiseptica* colonization in the LRT. Groups of 3–4 C57BL/6 mice were vaccinated with (A) 40 µg PRN, (B) 5 µg FHA or (C) 0.5 mL of 1∶5 dilution of Adacel in PBS and Imject Alum on Days 0 and 14. Adjuvant-only control (white bars) and protein with adjuvant (black bars) vaccinated mice were challenged on Day 28 with *B. pertussis* (*B. p.*) or *B. bronchiseptica* (*B. b.*) and dissected Day 3 post-challenge. Bacterial numbers are represented as the mean Log_10_ CFU+/−SEM. Dashed line indicates the lower limit of detection. * indicates statistical difference (*P* value of <0.05) between adjuvant only and protein with adjuvant treated groups.

Since pertactin and filamentous hemagglutinin are expressed by both *B. pertussis* and *B. bronchiseptica* and are included in some current acellular vaccines, we went on to determine if immunity to pertactin or filamentous hemagglutinin contributed to *B. pertussis*-induced immunity to *B. bronchiseptica*. Wild type mice were immunized with purified *B. pertussis*-derived pertactin, filamentous hemagglutinin, or aP vaccine, which contains these two shared antigens. These mice were then challenged with *B. pertussis* or *B. bronchiseptica* and dissected 3 days later to quantify bacterial numbers. Immunization reduced numbers of *B. pertussis* by>99%, relative to adjuvant-treated mice, (Figure 6A, B and C), consistent with previous findings [34]. Immunization with these *B. pertussis* antigens reduced *B. bronchiseptica* numbers by>90% in the trachea and lungs, as compared to numbers in adjuvant-treated mice (Figure 6). These data indicate that immunization with *B. pertussis*-derived antigens induces an immune response which protects the trachea and lungs of mice against either *B. pertussis* or *B. bronchiseptica* infection.

### *B. pertussis*-induced immunity protects against human isolates of *B. bronchiseptica*

**Figure 7:**
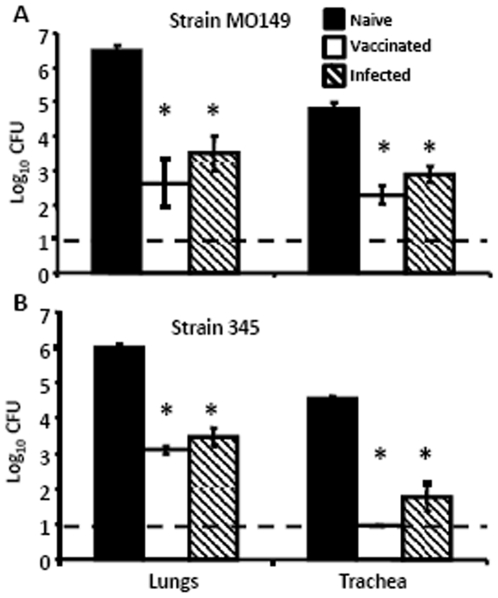
Human isolated, Complex IV *B. bronchiseptica* colonization of *B. pertussis*-immune mice. Groups of 4 naïve (black bars), *B. pertussis*-vaccinated (white bars) or *B. pertussis*-convalescent (hatched bars) C57BL/6 mice were challenged with *B. bronchiseptica* strain (A) M0149 or (B) 345 and dissected Day 3 post-challenge. Bacterial numbers are represented as the mean Log_10_ CFU+/−SEM. Dashed line indicates the lower limit of detection. * indicates statistical difference (*P* value of <0.05) between naïve and treated groups.

To determine if *B. pertussis*-induced immunity is sufficient to protect against recent human clinical isolates of *B. bronchiseptica*, wild type mice were immunized with *B. pertussis* and challenged with two *B. bronchiseptica* Complex IV isolates [5], strain M0149 and strain 345. These strains, which are divergent from the prototypical *B. bronchiseptica* Complex I strain, RB50, are more closely related to *B. pertussis* and are both from human-associated lineages [5]. Since the normal 5×10^5^ CFU dose was lethal to naïve mice, strain 345 was delivered in inocula of 2×10^5^ CFU. These bacteria grew to 10^6^ in the lungs and 10^5^ in the trachea of naïve mice (Figure 7). In contrast, *B. pertussis*-immunization reduced the numbers of both by greater than 99.9% in the lungs and trachea (Figure 7A and B) by Day 3 post-challenge; indicating that immunity to *B. pertussis* is sufficient to control *B. bronchiseptica* strains recently isolated from humans.

## Discussion

*B. parapertussis* avoids *B. pertussis*-induced immunity, a critical aspect of its ability to transmit efficiently to be successful in largely *B. pertussis*-immune human populations [19], [41]–[47] (X. Zhang, M. E. Rodríguez, E. T. Harvill, unpublished data). Although many *B. bronchiseptica* lineages have broad host ranges, infect large proportions of the animals in greatest contact with humans [3], [4], and are known to spillover from their animal hosts to humans [12], *B. bronchiseptica* strains are not observed to transmit amongst humans. Based on these observations we explored the possibility that *B. bronchiseptica* may be limited in its ability to infect humans because it cannot avoid *B. pertussis*-induced immunity. If *B. bronchiseptica* cannot avoid *B. pertussis*-induced immunity, then well-established ecological theory predicts that it cannot coexist with the human pathogen, explaining many of the observations described above.

Immunity induced by *B. pertussis* infection or vaccination significantly reduced *B. bronchiseptica* numbers in the LRT of mice (Figure 1). Although the mechanism of protection in the trachea and lungs appeared to be substantially similar, the major mucosal antibody, IgA, was not required for *B. pertussis*-induced protection against *B. bronchiseptica* in the lungs, but was important in the trachea (Figure 2). This is consistent with our recent observations that IgA is required for efficient control of *B. bronchiseptica* in the trachea but not the lungs [48]. Immune serum recognized *B. bronchiseptica* antigens and mediated protection that was dependent on complement and neutrophils, but immunization-induced immunity did not appear to require either complement or FcγRs (Figures 3, 4, and 5). Immunization with purified pertactin or filamentous hemagglutinin derived from *B. pertussis* also conferred protection against *B. bronchiseptica* (Figure 6). Together, these data indicate that *B. pertussis*-induced immunity provides protection against *B. bronchiseptica* in the LRT of mice.

In developed countries, vaccination against *B. pertussis* begins at a very early age. In certain populations, the seroprevalence of *B. pertussis*-specific antibodies exceeds 90% [49]. If the ability to colonize the LRT in large numbers and cause inflammation and disease symptoms (coughing) are important to *B. bronchiseptica* transmission, as they are to the other bordetellae, then these measures of immunity to *B. pertussis* are likely to limit the infectiousness of *B. bronchiseptica* in humans [17]. This “herd immunity” would not protect individuals in whom immunity had waned, or those that are immunodeficient, but the high prevalence of *B. pertussis*-immunity in their surroundings would dampen opportunities for transmission by decreasing the number of susceptible individuals. This would explain why *B. bronchiseptica* infections are generally associated with an animal source and why chains of transmission are generally not established [12]. Furthermore, this theoretical framework also explains the observed association of *B. bronchiseptica* disease in humans with an immunocompromised state [7]–[12], [50], [51].

Our data show that *B. pertussis* immunity does not protect the nasal cavities of mice from *B. bronchiseptica* colonization (Figure 1). It is possible, and consistent with our model, that *B. bronchiseptica* may be present in the nasal cavity of humans. However, it would be hard to imagine how either the bacterium or its immunological effects on *B. pertussis* epidemiology, and serological monitoring, could go unnoticed. Extensive samplings of nasophayngeal swabs and testing of serum reactivity that have revealed *B. pertussis*, *B. parapertussis* and even *B. holmesii* have not revealed *B. bronchiseptica* [3], [12], [42]–[44], [52]–[55], even though it is easier to culture. Alternatively, it is possible that the nasal cavity of humans is not conducive to *Bordetella* infection, possibly due to differences in physiology, immunology or other competitive flora. This could explain why *B. pertussis* appears to have lost the ability to persist in the nasal cavity of mice, while every *B. bronchiseptica* strain examined retains this ability [23], [56] (A.M. Buboltz, T.L. Nicholson, L.S. Weyrich, E.T. Harvill, unpublished data). However, since human *Bordetella* infections are highly contagious via the cough-induced respiratory droplets, the ability of *B. pertussis*-induced immunity to limit *B. bronchiseptica* to the nasal cavity should be sufficient to prevent its efficient transmission.

There are likely to be multiple factors that limit the success of an invading pathogen in a new host. Although spillover opportunity and individual host specificity are often considered, more often overlooked are the possible effects of resident flora on the success of the invader. Our data show that immunity to *B. pertussis* can have a substantial effect on *B. bronchiseptica* infection. Although a high level of individual immunity may prevent many initial infections or diseases, the variable immunity within human populations is more likely to allow occasional infections while having a more dramatic effect via “herd immunity” that inhibits the establishment of a successful chain of transmission. This model is consistent with much of the existing experimental and clinical data, including sporadic infections often associated with an immunocompromised state [8]–[10], [12], [50], [51]. It also leads to the prediction that *B. bronchiseptica* infections in humans may occur much more commonly than are reported, and improved surveillance may test this prediction. We can further predict that changes in the *B. pertussis* vaccination- or infection-induced immune status of human populations will affect the observed rate of zoonotic *B. bronchiseptica* disease and, more importantly, their opportunity to establish chains of infection to emerge as new infectious agents.
